# Bullying victimisation, coping, and self-harm among adolescents from diverse inner-city London schools: an accelerated cohort study

**DOI:** 10.1186/s13034-025-01015-y

**Published:** 2025-12-24

**Authors:** Emma Wilson-Lemoine, Rina Dutta, Colette Hirsch, Gemma Knowles, Stephanie Smith, Samantha Davis, Katie Chamberlain, Daniel Stanyon, Aisha Ofori, Alice Turner, Esther Putzgruber, Holly Crudgington, Vanessa Pinfold, Ulrich Reininghaus, Seeromanie Harding, Charlotte Gayer-Anderson, Craig Morgan

**Affiliations:** 1 Health Service & Population Research Department, Institute of Psychiatry, Psychology & Neuroscience, King’s College London, London, UK; 2 ESRC Centre for Society and Mental Health, King’s College London, London, SE5 8AF UK; 3 Department of Psychological Medicine, Institute of Psychiatry, Psychology & Neuroscience, King’s College London, London, UK; 4 South London and Maudsley NHS Foundation Trust, London, UK; 5 Centre for Evidence and Implementation, London, UK; 6 McPin Foundation, 7-17 Great Dover Street, London, UK; 7 Department of Public Mental Health, Central Institute of Mental Health, Medical Faculty Mannheim, University of Heidelberg, Mannheim, Germany; 8 Department of Population Health Sciences, School of Life Course and Population Sciences, Faculty of Life Sciences & Medicine, King’s College, London, UK

**Keywords:** Bullying, Coping, Self-harm, Adolescence

## Abstract

**Objective:**

This study aimed to explore longitudinal associations between bullying victimisation, coping, and self-harm among adolescents living in a diverse, densely-populated urban population in London, United Kingdom.

**Method:**

Data on bullying victimisation, dispositional use of four coping strategies (active, avoidant, distraction, support seeking), and self-harm were drawn from REACH (Resilience, Ethnicity and AdolesCent mental Health), an accelerated cohort study of adolescent mental health in South London. Data was available for 3,060 adolescents aged 11–14 years (*M*_age_ = 12.4, 50.6% girls, > 80% ethnic minority groups) who were followed up 12 months’ later. Models used a combination of inverse probability weights with multiple imputation. Results are presented as adjusted risk ratios (aRRs), with all analyses adjusting for baseline self-harm, sex, age, free school meals and ethnic group.

**Results:**

Bullying victimisation at baseline was associated with self-harm at one-year follow up (aRR 1.66). Avoidant (aRR 1.36) and distraction (aRR 1.20) coping were associated with increased risk of self-harm. Active (aRR 0.74) and support seeking (aRR 0.78) coping were associated with decreased risk. None of the coping strategies moderated the association between bullying victimisation and self-harm, and no clear sex differences were found for any results.

**Conclusions:**

Findings underline the importance of tackling bullying and promoting coping as part of a wider holistic approach to modifying the impact of adverse experiences. Among victims of bullying, future research should investigate situation-specific measures of coping, as this will better explain how young people cope specifically with this form of childhood adversity.

**Supplementary Information:**

The online version contains supplementary material available at 10.1186/s13034-025-01015-y.

## Introduction

Self-harm is a public health concern and one of the strongest risk factors for future suicide, including among adolescents [24]. From a theoretical perspective, self-harm has been described as a maladaptive coping strategy for regulating emotions and managing the negative affect that arises from adverse individual and social factors [11, 39, 51, 58, 67]. The causes of self-harm are complex, and likely to comprise a combination of distal (e.g., past experiences, static characteristics) and proximal (i.e., recent, immediate) risk factors [51]. Indeed, recent systematic reviews have identified risk factors that are interpersonal (e.g., bullying) and psychological—or intrapersonal—such as self-esteem, self-concept and coping [1, 23, 72]. At a broader level, self-harm risk may be heightened in deprived urban environments due to increased exposure to crime, unstable work or unemployment, poor housing quality, and social fragmentation [65].

Bullying, a form of peer victimisation, is described as aggressive, intentional actions carried out by one or more persons repeatedly and over time against a victim who cannot easily defend themselves [54], and has consistently been identified as a risk factor for self-harm in young people [25, 47, 72, 75]. Urban youth living in areas of deprivation may be at particular risk of bullying victimisation, given that low family income is a strong risk factor, and racial bullying is more prevalent in urban than rural environments [20, 28]. However, few studies have explored the impact of bullying victimisation in densely-populated urban populations of socio-economic and ethnic diversity.

In a recent cross-sectional study among a diverse population of London adolescents [76], being a victim of bullying led to a three-fold risk of self-harm, with risk strong for both girls and boys. However, these studies need to be replicated longitudinally, giving consideration to mediating and moderating factors [35], and should consider the risks and mechanisms among boys and girls, as there may be gender-specific pathways to self-injurious thoughts and behaviours [68, 75, 78].

Alongside interpersonal risk factors of self-harm, protective and harmful intrapersonal factors have also been identified. Coping is one such intrapersonal factor that describes the thoughts and actions people use to manage stressful internal and external demands they encounter in different life situations [43]. Such coping strategies may be problem-focused (i.e., changing one’s actions to handle a stressor) or emotion-focused (i.e., changing one’s interpretations of an event)—with some emotion-focused responses being adaptive (e.g., seeking help) and some maladaptive (e.g., denying the problem). According to Ayers et al. [4], coping strategies are operationalised multidimensionally in four higher-order factors: active (i.e., problem-focused, approach strategies), avoidance, distraction, support seeking. Theory suggests that maladaptive, avoidant coping responses are more likely when stressors are perceived as uncontrollable [43, 74]. Given that adolescents from urban environments may have greater exposure to uncontrollable stressors (e.g., crime, poverty), they may have developed a coping style that favours avoidance-type behaviors.

Poor coping skills are associated with negative mental health outcomes, including self-harm [46]. In a recent meta-analysis and narrative review [14], maladaptive coping strategies linked to child and adolescent psychopathology have included strategies of emotional suppression, avoidance and denial. Distraction coping may be protective in situations of low control or those that feel unalterable [15] while active coping (including problem solving) and seeking support have consistently found mental health benefits [10, 36, 66]. Self-harm is consistently associated with avoidant coping strategies [23, 38], as well as low problem solving—such as not seeking help—and high emotional coping, such as feeling worthless or unimportant [10, 16, 17].

Importantly, there is also a gap to investigate whether girls and boys differ in their choice of coping strategies and whether this influences associations with self-harm. For example, studies have found that avoidance coping may be more strongly associated with non-suicidal self-injury in girls compared with boys [80], and active coping more protective against suicidal behaviour for boys than girls [27]. One suggestion is that girls encounter more interpersonal stressors which are typically less controllable [27, 64]; they may therefore be more likely to engage in avoidance coping [13, 27].

Based on research, theory and lived experience testimony, self-harm involves a combination of complex preceding factors, including bullying and trouble managing distressing emotions, so there is also value in exploring how specific predictors may work together to increase or decrease the risk [1, 26]. Additionally, less is known about which factors may mediate or moderate the association between bullying victimisation and self-harm [35]. Therefore, exploring predictors (e.g., bullying, coping) and moderators (e.g., bullying and coping, sex and bullying, sex and coping) in the association with self-harm could help to better understand and identify whether particular young people are at heightened risk of self-harm, including those that have experienced bullying victimisation.

In the few studies that have investigated the interaction between bullying victimisation and coping on negative health outcomes, active coping styles have been shown to protect adolescents from the negative effects of bullying [77], while distraction may be helpful when the stressor feels unalterable [15] and support seeking may reduce suicidality [49].

In the present study, we aimed to build on our cross-sectional findings [76] and explore the longitudinal associations between bullying victimisation, coping and self-harm among adolescents living in a diverse, densely-populated urban population in London, United Kingdom. Our hypotheses are: (1) Bullying victimisation will be associated with increased risk of self-harm at 12-month follow up; (2a) Avoidance coping will be associated with increased risk of self-harm at 12-month follow up; (2b) Active, distraction and support seeking coping will be associated with decreased risk of self-harm at 12-month follow up. Additionally, we will test each of these four coping strategies as potential moderators in the longitudinal association between bullying victimisation and self-harm. All results were investigated across the total sample (boys and girls combined) and also stratified by sex.

## Methods

### Study design, procedure and participants

Data were drawn from the Resilience, Ethnicity and AdolesCent mental Health (REACH) study, an accelerated cohort study of adolescent mental health in two areas of London (UK): Southwark and Lambeth [41]. REACH has a well-developed youth involvement programme, including a youth advisory group who were integral to its study design [62]. At baseline, 4,353 participants from 12 schools took part in the REACH study by completing a questionnaire. The present paper works with a sub-set of the REACH study, a sample of 3,060 young people from 10 schools (see Wilson-Lemoine et al. [76]). At baseline, questions on self-harm were not administered in two (originally pilot) schools and these schools were excluded from analyses in the present paper. A description of those included vs. not included in the analysis sample has been reported elsewhere [76]. Time 1 questionnaires were completed between February 2016 and January 2018 and Time 2 follow up took place approximately one year later. All study procedures were approved by the Psychiatry, Nursing and Midwifery Research Ethics Subcommittee (PNM-RESC), King’s College London (ref:15/162320).

### Measures

#### Demographics

Socio-demographic information included age, sex, free school meals status (a proxy for household income) and ethnic group (using categories from the 2011 UK census;[52]). Some smaller groups were combined (e.g., Arab, Chinese), resulting in ten ethnic groups for analyses (see [40]).

#### Bullying victimisation

Four items were taken from the Revised Olweus Bully/Victim Questionnaire [55], capturing physical, verbal, relational and cyber bullying. Example item: “I was called mean names, was made fun of, or teased in a hurtful way” (verbal bullying). At Time 1 and Time 2, students were asked how often these situations occurred in the past 6 months. Responses were given on a five-point ordinal scale: 0 (*None*), 1 (*Once or twice*), 2 (*A few times a month*), 3 (*Once a week*), 4 (*A few times a week*). Based on recommendations in the literature [56], responses were dichotomized to create a global score, where 0 = *not bullied* and 1 = *bullied*. In line with the literature [70], a minimum threshold of 2 (*a few times a month*) or above was used to classify a participant as a victim of bullying. A composite measure of bullying was created (i.e., “any” bullying, if a respondent reported being bullied for at least one sub-type).

#### Coping

An adapted version of the Children’s Coping Strategies Checklist-R1 [4, 59] by Cline et al. [12] was used to capture dispositional coping (i.e., general styles of coping, rather than situation-specific). Twenty-six items (out of the original 52) assessed four types of coping: active (12 items), avoidance (6 items), distraction (4 items), support seeking (4 items). Example items include: “I think about which things are best to do to handle the problem” (active); “I try to ignore it” (avoidance); “I do something like video games or a hobby” (distraction); “I talk to someone who could help me solve the problem” (support seeking). Participants were asked how often they usually do each thing to solve the problem or make themselves feel better. Possible responses were: “Never”; “Sometimes”; “Often”; “Most of the time”. A summary score was created for each sub-scale, calculated as the mean of all items, with a higher score indicating greater use of that coping strategy. When calculating the mean for each sub-scale, it was required that 50% of the items were complete (not left blank or refused to answer).

#### Self-harm

Lifetime self-harm was measured with one item from the Development and Adolescent Wellbeing Assessment (DAWBA): “Over the whole of your lifetime, have you ever tried to harm or hurt yourself?” [22]. Possible responses were “Yes”; “No”; “Don’t want to answer”. At Time 2, self-harm was measured using one item of self-harm from the Clinical Interview Schedule (CIS-R; [44]), which additionally captured whether self-harm occurred in the past year.

### Statistical analyses

Inverse probability weights (IPW) were created to account for non-response bias and restore representativeness of the sample (*n* = 3,060) to the full REACH cohort eligible to take part (*n =* 4,945). Next, guided by the approach for handling missing data by the Centre for Longitudinal Studies [69], a combination of IPW and multiple imputation (i.e., IPW/MI) was used to manage missing data on all included variables. This created a full dataset of 3,060 participants at both time points. Hypotheses 1 (H1) and 2 (H2) were addressed using a mixed effect logistic regression that included the weight variable and Stata’s mi estimate and melogit commands. Risk ratios (RRs) were calculated from odds ratios using the marginalized delta method (nlcom command; [45]).

Cronbach’s alpha was calculated for each coping sub-scale on the weighted dataset using the package alphawgt [31], but prior to multiple imputation, due to incompatibility with Stata’s mi estimate package. Internal consistency at Time 1 was as follows: active (α = 0.90); avoidance (α = 0.74); distraction (α = 0.69), support seeking (α = 0.83).

Crude associations were first calculated between bullying victimisation and each of the coping strategies (all Time 1) with self-harm (Time 2) in a series of logistic regressions, using the IPW/MI dataset (see Model A, Table 3). These variables were then entered into one multivariable logistic regression (Model B). The final model (Model C) controlled for baseline self-harm and a priori confounding variables: sex, age, free school meals status, ethnic group and clustering within schools. These variables were chosen based on theory (including empirical studies in the academic literature) and clinical judgement due to being associated with the main exposure or outcome in similar studies [3, 6, 9, 18, 30, 37, 42]. Finally, to investigate in exploratory analyses whether findings for Hypotheses 1 and 2 would be similar or different between boys and girls, two interaction terms were fitted (i.e., sex and bullying, sex and coping), and all results were stratified by sex.

To investigate whether any of the coping strategies moderated the association between bullying victimisation and self-harm, an interaction term was created between bullying victimisation and each coping strategy, and added into four separate regression models. Drawing on the work of Aiken and West [2], the point estimate for bullying victimisation was calculated for participants at 1 standard deviation above and below the mean of each coping strategy. This provided an indication of how higher or lower levels of each coping strategy affected the association between bullying victimisation and self-harm. Taking this approach meant that the coping variables could remain as continuous independent predictors (rather than taking a dichotomised approach to the coping variable that loses some information), while getting estimates for bullying victimisation as a risk factor for self-harm at different levels of coping. Finally, to explore results between boys and girls, a three-way interaction term between bullying victimisation, coping and sex was added into the model, with results stratified by sex. All analyses were performed in Stata version 17.0.

## Results

In total, 1,697 (55.5%) participants in the analysis sample (*n* = 3,060 at Time 1) had complete data on bullying victimisation, coping and self-harm at all time points. Table S1–2 presents a comparison of characteristics for the responders with complete vs. incomplete data. All ethnic groups were more likely to have incomplete data for the bullying victimisation and self-harm variables compared with White British children, except for children in the Indian, Pakistani, Bangladeshi or Other Black groups. A combination of inverse probability weights and multiple imputation (IPW/MI) restored representativeness to the original REACH sample, which is representative of young people aged 11–14 years in the target population based on free school meals and ethnicity (see Table S3, which also includes reasons for non-participation and item missingness; and Table S4 for unweighted sample characteristics). Moreover, using IPW/MI enabled analyses to take place on the full analysis sample of 3,060 participants. Therefore, the Time 1 sample included 3,060 young people (M_age_ = 12.4 years, range 11–14; 50.6% girls; 23.6% on free school meals; >85% minority ethnic groups; see Table 1).

**Table 1:** Weighted sample characteristics, overall and by sex, on multiply imputed data

Sociodemographic information	Overall		Girls		Boys	
	*n* ^a^	%	*n* ^a^	%	*n* ^a^	%
Sex at birth		*n =* 3,060		*n =* 1,560		*n =* 1,500
Girls	1549	50.63	1560	100.00	N/A	N/A
Boys	1511	49.37	N/A	N/A	1500	100.00
Free school meals		*n =* 3,060		*n =* 1,560		*n =* 1,500
No	2338	76.39	1184	75.88	1154	76.92
Yes	722	23.61	376	24.12	346	23.08
Ethnic group		*n =* 3,060		*n =* 1,560		*n =* 1,500
Any other	209	6.83	100	6.42	109	7.26
Black African	786	25.70	411	26.34	38	2.50
Black Caribbean	495	16.19	260	16.70	235	15.66
Indian, Pakistani, Bangladeshi	122	3.99	71	4.53	52	3.44
Latin American	159	5.20	73	4.68	86	5.73
Mixed/multiple	171	5.60	91	5.86	80	5.34
Mixed White and Black	276	9.03	138	8.84	138	9.23
Non-British White	291	9.52	143	9.18	148	9.87
Other Black	94	3.07	59	3.79	35	2.33
White British	455	14.87	213	13.66	242	16.10
Year group		*n =* 3,060		*n =* 1,560		*n =* 1,500
Year 7	1114	36.39	575	36.89	538	35.88
Year 8	991	32.38	524	33.56	468	31.18
Year 9	955	31.22	461	29.55	494	32.94
Age		*n =* 3,060		*n =* 1,560		*n =* 1,500
11	642	20.97	353	22.63	289	19.26
12	1056	34.51	537	34.39	519	34.63
13	943	30.83	494	31.65	450	29.98
14	418	13.67	177	11.32	241	16.07
15	1	0.03	0	0.00	1	0.06

### Prevalence of self-harm, bullying victimisation and coping

Table 2 presents prevalence of self-harm and bullying victimisation at each time point. At Time 1, lifetime self-harm was reported by 16.9% of participants, and at Time 2, 15.5% of the sample reported past-year self-harm. Self-harm was 1.5 (Time 1) to 2.2 (Time 2) times higher in girls than boys. Bullying victimisation in the past six months was reported by 22.3% (Time 1) and 18.8% (Time 2) of participants, with prevalence slightly higher for girls than boys.

For general use of avoidance, active (Time 1 only) and support seeking coping strategies, mean scores were higher in girls than boys, while boys reported higher levels of distraction coping (see Table 2). Table S5 presents these results using the unweighted, complete case sample. Tables S6–7 report the correlation coefficients between the coping strategies at each time point in the weighted data. The multiply imputed dataset was not used due to incompatibility between the correlate function and the Stata mi estimate commands. Across all time points, there were medium to large correlations between all variables (*r*_=_ .34 – 0.67; see Table S6). However, VIF scores indicated no presence of multicollinearity.

**Table 2:** Prevalence of self-harm, bullying victimisation and coping (mean scores) at times 1–2, total sample and by sex

		Total		Girls		Boys		Girls v boys				
		*n*	%	*n*	%	*n*	%	Adjusted RR^a^		Lower CI		Upper CI
Self-harm (T1)	No	2,543	83.11	1,252	80.27	1,290	86.02	Reference^b^				
	Yes	517	16.89	308	19.73	210	13.98	1.45		1.03		1.86
Self-harm (T2)	No	2,587	84.54	1,246	79.90	1,339	89.30	Reference^b^				
	Yes	473	15.46	314	20.10	161	10.70	2.18		1.52		2.84
Bullying victimisation (T1)	No	2,377	77.67	1,192	76.43	1,184	78.93	Reference^c^				
	Yes	683	22.33	368	23.57	316	21.07	1.13		1.02		1.25
Bullying victimisation (T2)	No	2,484	81.17	1,244	79.76	1,239	82.62	Reference^c^				
	Yes	576	18.83	316	20.24	261	17.38	1.28		1.03		1.53

Coping variable		Total		Girls		Boys		Coefficient for sex in adjusted models				
		*m* ^d^		*m* ^d^		*m* ^d^		*B*	*SE*	*t*	*df*	*p*
Avoidance	Time 1	1.43		1.49		1.36		0.15	0.03	5.11	3336.4	< 0.001
	Time 2	1.42		1.52		1.31		0.19	0.03	5.84	1381.0	< 0.001
Active	Time 1	1.35		1.39		1.31		0.09	0.03	2.93	4712.0	0.003
	Time 2	1.34		1.36		1.32		0.02	0.03	0.64	1022.5	0.519
Distraction	Time 1	1.37		1.22		1.53		-0.30	0.05	-6.32	19782.3	< 0.001
	Time 2	1.29		1.11		1.47		-0.37	0.04	-8.75	3563.6	< 0.001
Support seeking	Time 1	1.11		1.21		1.00		0.21	0.05	4.30	19590.7	< 0.001
	Time 2	1.07		1.15		1.00		0.11	0.04	2.65	1589.6	0.008

### The main effects of bullying victimisation on self-harm

Supporting Hypothesis 1, Time 1 bullying victimisation was associated with Time 2 self-harm, in unadjusted (Model A) and fully adjusted models that controlled for coping (Model B) and the a priori confounding variables (see Model C, Table 3). In the fully adjusted model, victims of bullying were over 1.5 times more likely to report self-harm at follow-up compared with non-bullied peers (aRR 1.66, 95% CI [1.23, 2.08]). Table S9 presents results from the regressions using the complete case data, with similar findings to Table 3. Moreover, as shown in Supplementary Tables 8 and Table 3 (Model C), the risk estimate for bullying victimisation reduced once baseline self-harm was added into the regression models (i.e., from aRR 2.56 and 2.41 [Models A and B] to aRR 1.66 [Model C]).

### The main effects of four coping strategies on self-harm

Supporting Hypothesis 2a, use of Time 1 avoidance coping strategies was associated with Time 2 self-harm, in unadjusted and adjusted models (see Table 3). In the fully adjusted model, a risk ratio of 1.36 suggests that for each one unit increase in the avoidance coping score, the risk of Time 2 self-harm increased by 36%.

Supporting Hypothesis 2b, active and support seeking coping strategies were associated with a lower risk of self-harm in unadjusted and adjusted models. In the fully adjusted model, the risk of self-harm decreased by 26% (active) and 22% (support seeking) for each unit increase of these coping scores.

Contrary to hypothesised expectations, distraction coping was not associated with a reduced risk of self-harm. In the unadjusted, univariable logistic regression (i.e., Model A [see Table 3]), distraction coping neither increased nor decreased risk of self-harm (RR 0.99 [0.86, 1.12]). In the fully adjusted model, the risk ratio increased, indicating a possible small effect of distraction coping increased on risk of self-harm [aRR 1.20 [0.94, 1.45]).

When the four coping strategies were entered into the same model (e.g., Models B and C [see Table 3]), the estimates for avoidance and distraction coping showed a larger increased risk of self-harm, while the estimates for active and support seeking coping showed a larger decrease in risk of self-harm, compared with the univariable associations in Model A. Supplementary Table 8 presents information about the (small) changes in risk ratios for each coping strategy when other covariates were added into the model in a step-wise fashion. Table S9 present results of the regressions using the complete case data, with similar results to Table 3 (using IPW/MI).

**Table 3:** Logistic regressions exploring associations between T1 bullying victimisation, T1 coping and T2 self-harm (outcome in all models)

Variable	Model A^a^			Model B^b^			Model C^c^		
	RR	95% CI		aRR	95% CI		aRR	95% CI	
Bullying victimisation	2.56	2.05	3.08	2.41	1.90	2.92	1.66	1.23	2.08
Avoidance coping	1.22	1.01	1.42	1.55	1.20	1.91	1.36	0.96	1.76
Active coping	0.86	0.73	0.99	0.70	0.51	0.89	0.74	0.49	1.00
Distraction coping	0.99	0.86	1.12	1.04	0.87	1.21	1.20	0.94	1.45
Support seeking coping	0.84	0.73	0.95	0.83	0.68	0.99	0.78	0.62	0.95

### Exploratory: the role of sex

Table 4 extends analyses by providing results for girls and boys separately. First, an interaction between bullying victimisation and sex (*p* = 0.368) was added into the fully adjusted multivariable logistic regression (i.e., Model C, Table 3), with results then stratified by sex (see Table 4). Bullying victimisation (Time 1) was associated with self-harm (Time 2) for boys (aRR [1.91 [1.19, 2.63]) and girls (aRR 1.50 [0.95, 2.05]).

Next, interaction terms (coping and sex) were added to models for each coping variable (*p* = 0.421 to 0.967) and results then stratified. There was no clear evidence that risk estimates varied between girls and boys.

**Table 4:** Associations between T1 bullying victimisation, T1 coping and T2 self-harm (outcome), stratified by sex

Variable	Total (*n* = 3,060)			Girls (*n* = 1,560)			Boys (*n* = 1,500)			Interaction term *p* value
	aRR^a^	95% CI		aRR^a^	95% CI		aRR^a^	95% CI		
										Sex X bullying victimisation interaction
Bullying victimisation	1.66	1.23	2.08	1.50	0.95	2.05	1.91	1.19	2.63	0.368
										Sex X coping interaction
Avoidance coping	1.36	0.96	1.76	1.35	0.86	1.83	1.38	0.79	1.98	0.910
Active coping	0.74	0.49	1.00	0.74	0.43	1.05	0.77	0.37	1.17	0.897
Distraction coping	1.20	0.94	1.45	1.27	0.97	1.56	1.09	0.73	1.45	0.421
Support seeking coping	0.78	0.62	0.95	0.78	0.58	0.99	0.77	0.50	1.04	0.967

### The moderating effects of coping in the association between bullying victimisation and self-harm

There was no clear evidence to suggest that any of the coping strategies modified the association between bullying victimisation and self-harm. Irrespective of whether participants used lower or higher levels of avoidance (low: aRR 1.64, high: 1.62), distraction (low: aRR 1.64, high: 1.64), active (low: aRR 1.74, high: aRR 1.58) or support seeking (low: aRR 1.54, high: aRR 1.84) coping strategies, risk estimates for Time 1 bullying on Time 2 self-harm were largely unchanged (*p* values 0.589 − 0.985; see Table 5). Supplementary Table 10 presents these results for girls and boys, using a three-way interaction of bullying victimisation, coping and sex. The RR estimates showed no clear, consistent patterns in the results and CIs were wide and overlapping (all interaction *p* values > 0.200), suggesting there may not be a moderating effect of any coping strategies in the association between bullying victimisation and self-harm for boys or girls.

**Table 5:** Associations between time 1 bullying victimisation, time 1 coping and time 2 self-harm (outcome) with two-way interactions

	Low use of coping strategy			High use of coping strategy			Bullying X coping interaction term *p* value
	aRR^a^	95% CI		aRR^a^	95% CI		
Avoidance							
Bullying victimisation	1.64	1.03	2.24	1.62	1.08	2.16	0.985
Active							
Bullying victimisation	1.74	1.13	2.35	1.58	0.80	2.36	0.757
Distraction							
Bullying victimisation	1.64	1.06	2.22	1.64	1.00	2.35	0.966
Support seeking							
Bullying victimisation	1.54	0.98	2.10	1.84	1.03	2.64	0.589

## Discussion

This study provides unique findings on bullying victimisation, coping and self-harm in a diverse, inner-city cohort of adolescents. Supporting our first hypothesis, we found that victims of bullying were 1.7 times more likely to report self-harm one year later, after adjusting for previous self-harm, coping, sex, age, free school meals, and ethnicity. Our second hypothesis was partially supported, with avoidant coping increasing risk of self-harm and active and support seeking coping decreasing risk of self-harm. Contrary to the hypothesised direction, there was no evidence that distraction coping was associated with reduced risk of self-harm, but some suggestive evidence of a slight increased risk of self-harm. There was no clear evidence to suggest that any of the four coping strategies moderated the association between bullying victimisation and self-harm. There were no clear, consistent differences in risk of self-harm between girls and boys based on their reports of bullying victimisation or coping across any analyses.

### Comparison with previous literature

Strong associations between bullying victimisation and self-harm are consistent with previous research [25, 75, 76]. From a theoretical perspective, victims of bullying may self-harm as a maladaptive way to manage negative affect and communicate the distress arising from intra- and interpersonal difficulties such as bullying [50, 58, 73]. Risk estimates in the present paper are weaker than our cross-sectional baseline findings (aRR 1.66 [1.23, 2.08] vs. aRR 3.35 [2.89, 3.82]). Much of this reduction can be explained by the addition of controlling for baseline self-harm in fully adjusted models. Otherwise, our findings are consistent with other longitudinal studies. For example, Heerde and Hemphill [25] found an OR of 2.06, 95% CI [1.67, 2.53] for bullying victimisation and self-harm (without suicidal intent) in a meta-analysis with effect sizes from 33 samples with a longitudinal design. Moore et al. [47] found an OR of 1.65 [1.34, 2.02] for the association between bullying and NSSI. Importantly, results from the present study extend knowledge in this area to an understudied population; adolescents from an ethnically diverse area of South London with high levels of economic deprivation.

Our results for the main effect of coping on self-harm show partial support z for one of the earlier theories of coping which differentiated between active (approach) and passive or avoidant coping [7, 48]. Examples of active (approach) coping include taking practical steps to solve a specific problem, and this includes reaching out for support from others to solve a problem. The items within active and support seeking coping would be described as examples of active (approach) coping. These findings align with results from a critical review by Guerreiro et al. [23], who concluded that problem-focused coping strategies could be protective against self-harm. The authors also emphasised how emotion-focused responses, particularly avoidance, are more likely to amplify the risk for self-harm.

Avoidance, and to an extent distraction, are examples of passive/avoidant coping and have often been more closely linked to negative outcomes [57, 71]. Although distraction coping has been negatively correlated with some negative outcomes (e.g., depression; [21]), in the present study we did not find a protective effect for reducing the risk of self-harm—indeed, in the final adjusted model, higher levels of distraction coping appeared to increase risk of self-harm by a small amount. One explanation is that distraction from pain (mental or physical) may mask issues that need to be addressed, and Johnson [32] discussed how frequent use of distraction can alter the regions of the brain responsible for processing pain. It could be that distraction coping strategies may habituate one’s tolerance for pain, through desensitisation to the physical sensations; this is a risk when considering that tolerance to pain is a strong risk factor for future suicide. Within the interpersonal theory of suicidal behaviour, this is termed ‘acquired capability’ for suicide, as it is an ability humans develop through life experiences, such as childhood trauma or previous self-harm [33].

We did not find convincing evidence to suggest that any of the coping strategies moderated the association between bullying victimisation and self-harm. One explanation is our choice of coping measure. We used a dispositional measure of coping which asked young people how they cope with stress in general; rather than asking young people with experience of bullying victimisation about how they cope with this specific situation. It could be that a dispositional measure of coping fails to capture the nuances within a complex, sensitive area such as victimisation, where use of a coping strategy in one situation, for one person, may not be generalisable to other situations or forms of bullying victimisation [29]. This would align with the conceptualisation of coping by Lazarus and Folkman [43], who described coping as a dynamic process that fluctuates depending on the changing demands and appraisals of the situation.

For example, an active goal-oriented coping style may reduce risk of negative outcomes for no or low exposure to bullying behaviours but once bullying victimisation becomes more severe or chronic, people with high active goal-oriented coping styles report more symptoms of anxiety [61, 79]. In situations of low control and low power (e.g., cyberbullying, where the abuse is anonymous), it may be better to distance oneself from the situation, by blocking a bully on social media, for instance.

With this in mind, future research may be better directed to tackling questions that aim to understand how young people experience distress in a given moment, in a particular situation, using methods such as Ecological Momentary Assessment (EMA). These study designs could help inform triggers or precipitating factors for self-harm [63]. Or, with a sample of young people with experience of interpersonal difficulties such as bullying victimisation, EMA studies could provide insight into the ways in which young people cope with such situations in their daily lives [60].

### Methodological considerations

Findings should be considered alongside some methodological limitations. We may not have had a big enough sample to detect interaction effects in the three-way interactions, which future studies may wish to consider. As is common among cohort studies there was a high level of attrition [5, 8] with participants who did not take part at Time 2 differing from those who completed the survey. However, we followed the approach by UCL’s Centre for Longitudinal Studies [69] and used IPW/MI to restore representativeness of the sample back to the original target population.

We acknowledge that different instruments were used to measure self-harm at Time 1 (lifetime) and Time 2 (past-year). However, we do not believe this affected our main findings for the following reasons. First, given that the average age of onset for bullying is earlier than self-harm [19, 34, 53], self-harm is less likely to precede bullying. Second, in the fully adjusted models, we controlled for lifetime self-harm at Time 1. Finally, in a different study using the same dataset, we conducted sensitivity analyses that looked at the association between Time 1 bullying and Time 2 self-harm with and without the participants who reported lifetime self-harm at baseline. We found almost identical risk ratios in the fully adjusted models (i.e., with baseline self-harm, sex, age, ethnicity, free school meals, clustering by schools).

There are also limitations with our choice of coping measure. First, a dispositional measure of coping does not consider the wider context of children’s lives. For example, adopting a support seeking coping strategy may be protective against self-harm because participants have strong support networks and someone who can help them with general stressors. This was outside the remit of the paper but future research may wish to explore links between coping strategies and the wider socioenvironmental context. Second, some of the coping sub-scales (particularly distraction coping, with the lowest Cronbach alpha) contained gendered items that may have produced artificial gendered differences in the data. For example, items such as ‘play sport’, ‘exercise’, ‘gaming’ are activities that boys, more than girls, are socialised to do from a very young age. Moreover, some items may not adequately capture coping strategies relevant for young people from diverse racial and ethnic backgrounds. Future measures should ensure that suitable alternative activities are listed, with measurements developed in collaboration with a range of young people from different backgrounds.

### Implications

Our results provide some insight into the way that inter- and intrapersonal variables (i.e., bullying victimisation, coping, self-harm) may be associated with self-harm; individually and in conjunction. While the environmental factor (bullying) is associated with self-harm, this risk reduces once baseline self-harm, the coping variables, and the sociodemographic confounding variables are added into the model. This suggests that anti-bullying initiatives remain important, but that it is important to consider other psychosocial and environmental factors. Similarly, avoidance, active and support seeking coping were consistently associated with self-harm, suggesting that parents and educators should support children to bolster their confidence in using active and support seeking strategies to manage inter- and intrapersonal difficulties.

Moreover, our findings that the risk of bullying victimisation on self-harm remained (relatively) similar irrespective of low or high use of each coping strategy suggest that dispositional coping alone is not enough to reduce the risk of self-harm. Moreover, our findings have implications for researchers investigating similar topics. For example, to better understand the interaction between coping and an interpersonal risk factor such as bullying, researchers may benefit best from using measures of dispositional *and* situation-specific coping. This would better clarify whether there is scope or utility in providing children who experience bullying victimisation with adaptive coping strategies (e.g., talking to a trusted adult) that could foster better mental health outcomes.

## Conclusion

In summary, this study reports the longitudinal associations between bullying victimisation, coping and self-harm in a representative sample of inner-city adolescents. Findings reveal strong associations between bullying victimisation and self-harm. Additionally, avoidant and, tentatively, distraction coping were associated with increased risk of self-harm, while active and support seeking strategies reduced this risk. There was no clear evidence that any of the four forms of dispositional coping moderated the association between bullying victimisation and self-harm, nor any clear differences in our findings between girls and boys. Future research should investigate situation-specific measures of coping, as this will better explain how young people cope specifically with being a victim of bullying; and how this may be associated with self-harm.

## Supplementary Information

Below is the link to the electronic supplementary material.

Supplementary Material 1

## Data Availability

C.M. had full access to all the data in the study and takes responsibility for the integrity of the data and the accuracy of the data analysis.
