# Supplementary material for: Bullying victimisation, coping, and self-harm among adolescents from diverse inner-city London schools: an accelerated cohort study
Source: Child Adolesc Psychiatry Ment Health. 2025 Dec 24;20:11. doi: 10.1186/s13034-025-01015-y (PMC12849072; doi:10.1186/s13034-025-01015-y)
Supplement: Supplementary file 1 — Supplementary Material 1 [file 13034_2025_1015_MOESM1_ESM.docx]

Supplementary materials to Wilson-Lemoine et al., 2025 “Bullying victimization, coping and self-harm among adolescents from diverse inner-city schools: an accelerated cohort study”

Table S1. Comparison of responders vs. non-responders to the bullying, coping and self-harm questions at Times 1 and 2 (categorical variables)

| Variable | Description | *n* | Not complete data on bullying, coping and self-harm^a^ (*n* = 1,363) | Complete data on bullying, coping and self-harm (*n* = 1,697) | χ^2^ | *p* |
| --- | --- | --- | --- | --- | --- | --- |
| Child sex | Female | 1,560 | 671 (49.23%) | 889 (52.39%) | 3.02 | .083 |
|  | Male | 1,500 | 692 (50.77%) | 808 (47.61%) |  |  |
| Free school meals (T1) | Yes | 560 | 283 (22.42%) | 277 (17.04%) | 13.20 | < .001 |
|  | No | 2,328 | 979 (77.58%) | 1,349 (82.96%) |  |  |
| Ethnic group | Black African | 794 | 356 (26.12%) | 438 (25.81%) | 28.40 | .001 |
|  | Black Caribbean | 414 | 201 (14.75%) | 213 (12.55%) |  |  |
|  | Indian, Pakistani, Bangladeshi | 140 | 55 (4.04%) | 85 (5.01%) |  |  |
|  | Latin American | 127 | 68 (4.99%) | 59 (3.48%) |  |  |
|  | Mixed White and Black | 272 | 132 (9.68%) | 140 (8.25%) |  |  |
|  | Mixed/multiple | 192 | 87 (6.38%) | 105 (6.19%) |  |  |
|  | Non-British White | 281 | 125 (9.17%) | 156 (9.19%) |  |  |
|  | Other (any other) | 203 | 102 (7.48%) | 101 (5.95%) |  |  |
|  | Other Black | 95 | 41 (3.01%) | 54 (3.18%) |  |  |
|  | White British | 542 | 196 (14.38%) | 346 (20.39%) |  |  |
| Age (T1) | 11 | 647 | 277 (20.32%) | 370 (21.80%) | 7.63 | .106 |
|  | 12 | 1,045 | 480 (35.22%) | 565 (33.29%) |  |  |
|  | 13 | 949 | 440 (32.28%) | 509 (29.99%) |  |  |
|  | 14 | 418 | 166 (12.18%) | 252 (14.85%) |  |  |
|  | 15 | 1 | 0 | 1 (0.06) |  |  |
| Cohort^b^ | Cohort 1 | 1,116 | 488 (35.80%) | 628 (37.01%) | 0.475 | .789 |
|  | Cohort 2 | 979 | 441 (32.36%) | 540 (31.82%) |  |  |
|  | Cohort 3 | 965 | 434 (31.84%) | 529 (31.17%) |  |  |

*Note.*

^a^Includes those who took part at Time 1 and were part of the analysis sample (*n* = 3,060) but did not have complete data on the variables of interest (i.e., bullying, coping and self-harm) at all time points (Times 1 and 2).

^b^Corresponds to the original cohort students were assigned to at Time 1 (e.g., Year 7 = Cohort 1, Year 8 = Cohort 2, Year 9 = Cohort 3).

Table S2. Comparison of responders vs. non-responders to the bullying, coping and self-harm questions at Times 1 and 2 (continuous)

| Variable | Not complete data on bullying and self-harm^a^ | | | Complete data on bullying and self-harm | | | Test statistics | | |
| --- | --- | --- | --- | --- | --- | --- | --- | --- | --- |
|  | *n* | *M* | *SD* | *n* | *M* | *SD* | *t* | *df* | *p* |
| SDQ - Internalising | 1,347 | 5.55 | 3.41 | 1,689 | 4.97 | 3.22 | 4.83 | 3034 | <. 001 |
| SDQ - Externalising | 1,350 | 6.81 | 3.61 | 1,689 | 5.83 | 3.54 | 7.55 | 3037 | < .001 |
| SMFQ (Depression) | 1,202 | 5.46 | 6.21 | 1,626 | 4.38 | 5.49 | 4.90 | 2826 | < .001 |

*Note.* SDQ = Strengths and Difficulties Questionnaire. SMFQ = Short Mood and Feelings Questionnaire.

^a^Includes those who took part at Time 1 and were part of the analysis sample (*n* = 3,060) but did not have complete data on the variables of interest (i.e., bullying, coping and self-harm) at all time points (Times 1 and 2).

**Table S3.** Sample characteristics of analysis sample vs. full REACH samples at Time 1

| Variable | Analysis sample (actual) | | Analysis sample (IPW only) | | Analysis sample (MI only) | | Analysis sample (IPW/MI) | | Participated at Time 1 (10 schools)^a^ | | Participated at Time 1 (12 schools)^b^ | |
| --- | --- | --- | --- | --- | --- | --- | --- | --- | --- | --- | --- | --- |
|  | (*n =* 3,060) | | (*n =* 3,060) | | (*n =* 3,060) | | (*n =* 3,060) | | (*n =* 3,537) | | (*n =* 4,353) | |
|  | n | % | n, calibrated | % | n | % | n, calibrated | % | n | % | n | % |
| Sex at birth | *n =* 3,060 | | *n =* 3,060 | | *n =* 3,060 | | *n =* 3,060 | | *n =* 3,537 | | *n =* 4,353 | |
| Boys | 1,500 | 49.02 | 1,511 | 49.37 | 1,500 | 49.02 | 1,511 | 49.37 | 1,713 | 48.43 | 2,140 | 49.16 |
| Girls | 1,560 | 50.98 | 1,549 | 50.63 | 1,560 | 50.98 | 1,549 | 50.63 | 1,824 | 51.57 | 2,213 | 50.84 |
| Free School Meals | *n =* 2,888^c^ | | *n =* 2,888 | | *n =* 3,060 | | *n =* 3,060 | | *n =* 3,325 | | *n =* 4,113 | |
| No | 2,328 | 80.61 | 2,210 | 76.52 | 2,458 | 80.31 | 2,338 | 76.39 | 2,666 | 80.18 | 3,136 | 76.25 |
| Yes | 560 | 19.39 | 678 | 23.48 | 602 | 19.69 | 722 | 23.61 | 659 | 19.82 | 977 | 23.75 |
| Ethnic group | *n =* 3,060 | | *n =* 3,060 | | *n =* 3,060 | | *n =* 3,060 | | *n =* 3,537 | | *n =* 4,353 | |
| Black African | 794 | 25.95 | 786 | 25.70 | 794 | 25.95 | 786 | 25.70 | 926 | 26.18 | 1112 | 25.55 |
| Black Caribbean | 414 | 13.53 | 495 | 16.19 | 414 | 13.53 | 495 | 16.19 | 499 | 14.11 | 714 | 16.40 |
| Indian, Pakistani, Bangladeshi | 140 | 4.58 | 122 | 3.99 | 140 | 4.58 | 122 | 3.99 | 152 | 4.30 | 180 | 4.14 |
| Latin American | 127 | 4.15 | 159 | 5.20 | 127 | 4.15 | 159 | 5.20 | 154 | 4.35 | 220 | 5.05 |
| Mixed White and Black | 272 | 8.89 | 276 | 9.03 | 272 | 8.89 | 276 | 9.03 | 315 | 8.91 | 386 | 8.87 |
| Mixed/multiple | 192 | 6.27 | 171 | 5.60 | 192 | 6.27 | 171 | 5.60 | 215 | 6.08 | 243 | 5.58 |
| Non-British White | 281 | 9.18 | 291 | 9.52 | 281 | 9.18 | 291 | 9.52 | 324 | 9.16 | 430 | 9.88 |
| Other (any other) | 203 | 6.63 | 209 | 6.83 | 203 | 6.63 | 209 | 6.83 | 241 | 6.81 | 299 | 6.87 |
| Other Black | 95 | 3.1 | 94 | 3.07 | 95 | 3.10 | 94 | 3.07 | 109 | 3.08 | 130 | 2.99 |
| White British | 542 | 17.71 | 455 | 14.87 | 542 | 17.71 | 455 | 14.87 | 602 | 17.02 | 639 | 14.68 |
| Year Group | *n =* 3,060 | | *n =* 3,060 | | *n =* 3,060 | | *n =* 3,060 | | *n =* 3,537 | | *n =* 4,353 | |
| Year 7 | 1,116 | 36.47 | 1,114 | 36.39 | 1,116 | 36.47 | 1,114 | 36.39 | 1,329 | 37.57 | 1,593 | 36.60 |
| Year 8 | 981 | 32.06 | 991 | 32.38 | 981 | 32.06 | 991 | 32.38 | 1,139 | 32.20 | 1,423 | 32.69 |
| Year 9 | 963 | 31.47 | 955 | 31.22 | 963 | 31.47 | 955 | 31.22 | 1,069 | 30.22 | 1,337 | 30.71 |
| Age | *n =* 3,060 | | *n =* 3,060 | | *n =* 3,060 | | *n =* 3,060 | | *n =* 3,537 | | *n =* 4,353 | |
| 11 | 647 | 21.14 | 642 | 20.97 | 647 | 21.14 | 642 | 20.97 | 770 | 21.77 | 908 | 20.86 |
| 12 | 1045 | 34.15 | 1056 | 34.51 | 1045 | 34.15 | 1056 | 34.51 | 1,225 | 34.63 | 1,493 | 34.30 |
| 13 | 949 | 31.01 | 943 | 30.83 | 949 | 31.01 | 943 | 30.83 | 1,078 | 30.48 | 1,357 | 31.17 |
| 14 | 418 | 13.66 | 418 | 13.67 | 418 | 13.66 | 418 | 13.67 | 463 | 13.09 | 594 | 13.65 |
| 15 | 1 | 0.03 | 1 | 0.03 | 1 | 0.03 | 1 | 0.03 | 1 | 0.03 | 1 | 0.02 |
| SELF-HARM | n | % | n, calibrated | % | n | % | n, calibrated | % | n | % | n | % |
| Lifetime self-harm | *n =* 3,060 | | *n =* 3,060 | | *n =* 3,060 | | *n =* 3,060 | | *n =* 3,060 | | *n =* 3,061 | |
| No | 2,619 | 85.59 | 2,543 | 83.11 | 2,619 | 85.59 | 2,543 | 83.11 | 2,619 | 85.59 | 2,620 | 85.59 |
| Yes, ever | 441 | 14.41 | 517 | 16.89 | 441 | 14.41 | 517 | 16.89 | 441 | 14.41 | 441 | 14.41 |
| BULLYING VICTIMISATION | n | % | n, calibrated | % | n | % | n, calibrated | % | n | % | n | % |
| Bully victim (any type) | *n =* 2,896^d^ | | *n =* 2,896 | | *n =* 3,060 | | *n =* 3,060 | | *n =* 3,219 | | *n =* 3,923 | |
| No | 2,341 | 80.84 | 2,259 | 77.99 | 2,465 | 80.54 | 2,377 | 77.67 | 2,560 | 79.53 | 3,126 | 79.68 |
| Yes | 555 | 19.16 | 637 | 22.01 | 595 | 19.46 | 683 | 22.33 | 659 | 20.47 | 797 | 20.32 |

Notes. IPW = Inverse probability weights. MI = Multiple imputation. IPW/MI = combination of IPW and MI.

^a^Sample includes anyone who completed a T1 questionnaire, from 10 schools (i.e., excluding the 2 pilot schools that did not ask about self-harm). Originally 4,021 participants from the 10 schools were eligible to take part. Of this number, reasons for non-participation: absent (*n =* 284, 7.06%); non-assent (*n =* 46; 1.14%); opt out (*n =* 145, 3.61%); technical issues (*n =* 9; 0.22%).

^b^Sample includes anyone who completed a T1 questionnaire. Originally 4,945 participants from the 12 schools were eligible to take part. Of this number, reasons for non-participation: absent (*n =* 353, 7.14%); non-assent (*n =* 57; 1.15%); opt out (*n =* 167, 3.38%); technical issues (*n =* 15; 0.30%)

^c^Reasons for missing data: skipped or did not get to this point in the questionnaire (*n =* 3; 0.10%); did not know (*n =* 155; 5.07%); refused to answer this question (*n =* 14; 0.46%)

^d^ Reasons for missing data: skipped or did not get to this point in the questionnaire (*n =* 85; 2.78%); refused to answer this question (*n =* 79; 2.58%)

**Table S4.** Raw sample characteristics in the analysis sample (*n =* 3,060), total and by sex, unweighted and no multiple imputation

| Variable | Total | | Girls | | Boys | | Test for group differences |
| --- | --- | --- | --- | --- | --- | --- | --- |
|  | *n* | *%* | *n* | *%* | *n* | *%* | *x*^2^ |
| Sex | *n =* 3,060 | | *n =* 1,560 | | *n =* 1,500 | |  |
| Girls | 1560 | 50.98 | 1560 | 100.00 | -- | -- | -- |
| Boys | 1500 | 49.02 | -- | -- | 1,500 | 100.00 |  |
| Free School Meals | *n =* 2,888 | | *n* = 1,470 | | *n =* 1,418 | | *x*^2^(2) = 1.73, *p* = .189 |
| No | 2328 | 80.61 | 1171 | 79.66 | 1157 | 81.59 |  |
| Yes | 560 | 19.39 | 299 | 20.34 | 261 | 18.41 |  |
| Ethnic group | *n =* 3,060 | | *n =* 1,560 | | *n =* 1,500 | | *x*^2^(9) = 14.10, p = .119 |
| Black African | 794 | 25.95 | 413 | 26.47 | 381 | 25.40 |  |
| Black Caribbean | 414 | 13.53 | 217 | 13.91 | 197 | 13.13 |  |
| Indian, Pakistani, Bangladeshi | 140 | 4.58 | 82 | 5.26 | 58 | 3.87 |  |
| Latin American | 127 | 4.15 | 61 | 3.91 | 66 | 4.40 |  |
| Mixed White and Black | 272 | 8.89 | 136 | 8.72 | 136 | 9.07 |  |
| Mixed/multiple | 192 | 6.27 | 102 | 6.54 | 90 | 6.00 |  |
| Any Other | 203 | 6.63 | 97 | 6.22 | 106 | 7.07 |  |
| Other Black | 95 | 3.10 | 59 | 3.78 | 36 | 2.40 |  |
| Mixed White and Black | 272 | 8.89 | 136 | 8.72 | 136 | 9.07 |  |
| White British | 542 | 17.71 | 255 | 16.35 | 287 | 19.13 |  |
| Year Group | *n =* 3,060 | | *n* = 1,560 | | *n =* 1,500 | | *x*^2^(2) = 5.96, p = .051 |
| Year 7 | 1,116 | 36.47 | 581 | 37.24 | 535 | 35.67 |  |
| Year 8 | 981 | 32.06 | 519 | 33.27 | 462 | 30.80 |  |
| Year 9 | 963 | 31.47 | 460 | 29.49 | 503 | 33.53 |  |
| Age (*M*_age_ = 12.37, *SE* = 0.02) | *n =* 3,060 | | *n =* 1,560 | | *n =* 1,500 | | x2(4) = 20.37, p < .001 |
| 11 | 647 | 21.14 | 358 | 22.95 | 289 | 19.27 |  |
| 12 | 1045 | 34.15 | 532 | 34.10 | 513 | 34.20 |  |
| 13 | 949 | 31.01 | 495 | 31.73 | 454 | 30.27 |  |
| 14 | 418 | 13.66 | 175 | 11.22 | 243 | 16.20 |  |
| 15 | 1 | 0.03 | 0 | 0.00 | 1 | 0.07 |  |

Table S5. Prevalence of self-harm, bullying and coping (mean scores) at Times 1-3, total sample and by sex, unweighted complete case data

|  |  | | Total | | Girls | | Boys | | Girls v Boys | | | | | |  |
| --- | --- | --- | --- | --- | --- | --- | --- | --- | --- | --- | --- | --- | --- | --- | --- |
|  |  | | *n* | % | *n* | % | *n* | % | Chi^2^ | | *df* | | *p* | |  |
| Self-harm (T1) | No | 2,619 | | 85.59 | 1,304 | 83.59 | 1,315 | 87.67 |  | | | | | |  |
|  | Yes | 441 | | 14.41 | 256 | 16.41 | 185 | 12.33 | 10.305 | | 1 | | .001 | |  |
| Self-harm (T2) | No | 2,075 | | 88.45 | 1,034 | 85.31 | 1,041 | 91.80 |  | | | | | |  |
|  | Yes | 271 | | 11.55 | 178 | 14.69 | 93 | 8.20 | 24.117 | | 1 | | < .001 | |  |
| Bullying (T1) | No | 2,341 | | 80.84 | 1,196 | 79.89 | 1,145 | 81.84 |  | | | | | |  |
|  | Yes | 555 | | 19.16 | 301 | 20.11 | 254 | 18.16 | 1.777 | | 1 | | .183 | |  |
| Bullying (T2) | No | 2,037 | | 84.17 | 1,064 | 83.45 | 973 | 84.98 |  | | | | | |  |
|  | Yes | 383 | | 15.83 | 211 | 16.55 | 172 | 15.02 | 1.056 | | 1 | | .304 | |  |
| Coping variable |  | | Total | | Girls | | Boys | | Coefficient for sex in unadjusted models | | | | | |  |
|  |  | | *m (SD)* | | *m (SD)* | | *m (SD)* | | *t* | *df* | | *p* | | *d* | |
| Avoidance | Time 1 | | 1.42 (0.69) | | 1.48 (0.68) | | 1.35 (0.70) | | -4.88 | 2617 | | < .001 | | -0.19 | |
|  | Time 2 | | 1.42 (0.66) | | 1.52 (0.62) | | 1.32 (0.69) | | -7.23 | 2181 | | < .001 | | -0.31 | |
| Active | Time 1 | | 1.37 (0.71) | | 1.41 (0.69) | | 1.34 (0.72) | | -2.57 | 2559 | | .010 | | -0.10 | |
|  | Time 2 | | 1.37 (0.70) | | 1.39 (0.64) | | 1.34 (0.76) | | -1.49 | 2163 | | .137 | | -0.06 | |
| Distraction | Time 1 | | 1.37 (0.79) | | 1.22 (0.80) | | 1.53 (0.75) | | 10.16 | 2578 | | < .001 | | 0.40 | |
|  | Time 2 | | 1.29 (0.78) | | 1.11 (0.74) | | 1.48 (0.78) | | 11.14 | 2172 | | < .001 | | 0.48 | |
| Support seeking | Time 1 | | 1.13 (0.84) | | 1.24 (0.86) | | 1.02 (0.80) | | -6.81 | 2566 | | < .001 | | -0.27 | |
|  | Time 2 | | 1.11 (0.83) | | 1.18 (0.82) | | 1.02 (0.84) | | -4.59 | 2164 | | < .001 | | -0.20 | |

*Note*. CI = 95% Confidence Interval. *B* = unstandardised beta coefficient. RR = Risk ratio.

Table S6. Correlation matrix for coping (mean scores) at Time 1 using weighted data

|  | 1 | 2 | 3 | 4 |
| --- | --- | --- | --- | --- |
| 1. Avoidance^a^ | 1.00 |  |  |  |
| 2. Active^b^ | .67*** | 1.00 |  |  |
| 3. Distraction^c^ | .35*** | .47*** | 1.00 |  |
| 4. Support seeking^d^ | .43*** | .61*** | .34*** | 1.00 |

*Note*. Results presented using Pearson *r* correlation coefficient. Results represent the Time 1 weighted sample without multiple imputation (*n* = 3,060) as it is unsupported using the Stata mi estimate command. *** Correlation coefficient is significant at the 0.01 level (2-tailed).

^a^Item response: *n* = 2,619. ^b^Item response: *n* = 2,561. ^c^Item response: *n* = 2,580. ^d^Item response: *n* = 2,568.

Table S7. Correlation matrix for coping (mean scores) at Time 2 using weighted data

|  | 1 | 2 | 3 | 4 |
| --- | --- | --- | --- | --- |
| 1. Avoidance^a^ | 1.00 |  |  |  |
| 2. Active^b^ | .67*** | 1.00 |  |  |
| 3. Distraction^c^ | .34*** | .47*** | 1.00 |  |
| 4. Support seeking^d^ | .41*** | .60*** | .36*** | 1.00 |

*Note*. Results presented using Pearson *r* correlation coefficient. Results represent the Time 2 weighted sample without multiple imputation (*n* = 2,637) as it is unsupported using the Stata mi estimate command. *** Correlation coefficient is significant at the 0.01 level (2-tailed).

^a^Item response: *n* = 2,183. ^b^Item response: *n* = 2,165. ^c^Item response: *n* = 2,174. ^d^Item response: *n* = 2,166.

**Table S8.** Stepwise associations between bullying (Time 1), coping (Time 1) and self-harm (Time 2), with covariate estimates, using the IPW/MI dataset

|  |  |  |  | Risk Ratios (95% CI) | |  |  |  |
| --- | --- | --- | --- | --- | --- | --- | --- | --- |
|  | Model 1a | Model 1b | Model 2 | Model 3 | Model 4 | Model 5 | Model 6 | Model 7 |
| Bullying | 2.56  [2.05,3.08] | 2.41  [1.90,2.93] | 2.42  [2.00,2.84] | 1.64  [1.24,2.04] | 1.64  [1.20,2.07] | 1.66 [1.23,2.08] | 1.66  [1.23,2.08] | 1.66  [1.23,2.08] |
| Avoidance coping | - | 1.55  [1.20,1.91] | 1.54  [1.09,1.91] | 1.43  [1.02,1.85] | 1.36  [0.97,1.76] | 1.36  [0.97,1.76] | 1.36  [0.96,1.76] | 1.36  [0.96,1.76] |
| Active coping | - | 0.70  [0.51,0.89] | 0.70  [0.52,0.88] | 0.77  [0.53,1.02] | 0.75  [0.51,1.00] | 0.74  0.49, 1.00] | 0.74  [0.49,0.99] | 0.74  [0.49,1.00] |
| Distraction coping | - | 1.04  [0.87,1.21] | 1.05  [0.87,1.24] | 1.04  [0.83,1.25] | 1.18  [0.94, 1.43] | 1.20 [0.95,1.45] | 1.20  [0.95,1.45] | 1.20  [0.94,1.45] |
| Support seeking coping (T1) | - | 0.83  [0.69,0.99] | 0.83  [0.67,0.98] | 0.83  [0.66,1.01] | 0.78  [0.62,0.95] | 0.78  [0.62,0.95] | 0.78  [0.62,0.95] | 0.78  [0.62,0.95] |
| Self-harm (T1) | - | - | - | 6.77  [4.56, 8.99] | 8.00  [5.19,10.82] | 8.24 [5.63,10.84] | 8.21  [5.58,10.83] | 8.10  [5.46,10.73] |
| Sex | - | - | - | - | 2.39  1.48,3.31] | 2.44  [1.53, 3.34] | 2.42  [1.52,3.33] | 2.42  [1.52,3.23] |
| Age (T1) | - | - | - | - | - | 1.09 [0.83,1.34] | 1.08  [0.83,1.34] | 1.08  [0.83,1.34] |
| FSM (T1) | - | - | - | - | - | - | 0.87  [0.58,1.16] | 0.87  [0.58,1.17] |
| Ethnicity | - | - | - | - | - | - | - | 0.99  [0.95,1.02] |

*Note*. IPW/MI = Using inverse probability weights and multiply imputed data. Models 1a and 1b are unadjusted. Models 2 – 7 are adjusted for clustering by school. Model 3 is adjusted for baseline self-harm. Model 4 is also adjusted for sex. Model 5 is also adjusted for age. Model 6 is also adjusted for free school meals. Model 7 is adjusted for all explanatory variables under investigation and potential confounders in the same model.

Table S9. A series of univariable logistic regressions and a multiple logistic regression, adjusted for potential a priori confounders, exploring longitudinal associations between T1 bullying, T1 coping and T2 self-harm, using complete case data

| Variable |  | Model A^a^  (*n* = 1,950 - 2,238) | | |  | Model B^b^  (*n* = 1,950) | | |  | Model C^c^  (*n* = 1,862) | | |
| --- | --- | --- | --- | --- | --- | --- | --- | --- | --- | --- | --- | --- |
|  |  | RR | 95% CI | |  | aRR | 95% CI | |  | aRR | 95% CI | |
| Bullying |  | 2.67 | 2.07 | 3.28 |  | 2.60 | 1.95 | 3.24 |  | 1.79 | 1.16 | 2.42 |
| Avoidance coping |  | 1.20 | 0.98 | 1.42 |  | 1.56 | 1.17 | 1.95 |  | 1.31 | 0.90 | 1.71 |
| Active coping |  | 0.82 | 0.68 | 0.95 |  | 0.69 | 0.49 | 0.90 |  | 0.74 | 0.47 | 1.00 |
| Distraction coping |  | 0.98 | 0.83 | 1.13 |  | 1.07 | 0.88 | 1.27 |  | 1.17 | 0.90 | 1.45 |
| Support seeking coping |  | 0.77 | 0.66 | 0.88 |  | 0.75 | 0.60 | 0.90 |  | 0.77 | 058 | 0.95 |

*Note.* Results using complete case dataset. CI = 95% Confidence Interval. RR = Risk ratio.

^a^Unadjusted, univariable associations

^b^Multivariable logistic regression with main explanatory variables under investigation (bullying and the four coping strategies)

^c^As Model B, but RRs additionally adjusted for baseline self-harm, sex, age, free school meals, ethnic group

**Table S10.** Longitudinal associations between Time 1 bullying, Time 1 coping and Time 2 self-harm with three-way interactions

|  | Low use of coping strategy | | | High use of coping strategy | | | Bullying*Coping*Sex interaction term *p-*value |
| --- | --- | --- | --- | --- | --- | --- | --- |
|  | aRR^a^ | 95% CI | | aRR^a^ | 95% CI | |  |
| Model 1: Avoidance |  |  |  |  |  |  | .947 |
| Bullying for girls | 1.55 | 0.50 | 2.60 | 1.67 | 0.99 | 2.34 |  |
| Bullying for boys | 1.93 | 0.85 | 3.00 | 1.98 | 0.95 | 3.02 |  |
| Model 2: Active |  |  |  |  |  |  | .255 |
| Bullying for girls | 1.90 | 1.03 | 2.77 | 1.38 | 0.60 | 2.16 |  |
| Bullying for boys | 1.70 | 0.71 | 2.69 | 2.42 | 0.92 | 3.92 |  |
| Model 3: Distraction |  |  |  |  |  |  | .595 |
| Bullying for girls | 1.69 | 0.94 | 2.44 | 1.53 | 0.65 | 2.40 |  |
| Bullying for boys | 1.74 | 0.56 | 2.92 | 2.16 | 1.14 | 3.18 |  |
| Model 4: Support seeking | |  |  |  |  |  | .885 |
| Bullying for girls | 1.47 | 0.69 | 2.24 | 1.80 | 0.78 | 2.82 |  |
| Bullying for boys | 1.78 | 0.82 | 2.74 | 2.40 | 0.81 | 3.99 |  |

*Note.* IPW/MI (combination of inverse probability weights and multiple imputation) was used for these analyses. CI = 95% Confidence Interval. aRR = adjusted risk ratio.

^a^aRR, adjusted for baseline self-harm, sex, age, free school meals, ethnic group and clustering by schools.
